# Statistics of 109 London Hospitals in 1915

**Published:** 1916-11-25

**Authors:** 


### REPORT OF KING EDWARD'S HOSPITAL FUND.

### Statistics of 109 London Hospitals in 1915.

It will be remembered that the issue of the Statistical Report for 1914 was regarded as the first of a new or transitional series. For the first time the general hospitals with medical schools were separated in the tables from the other larger general hospitals, and as a temporary measure comparisons of cost per bed between the current and the previous year were omitted. These features continue in the 1915 Report, but the invitation for further suggestions of alterations in the methods of presenting the statistics which appeared in Mr. Maynard's preface to the 1914 volume has apparently not led to any further innovations. Chiefly for reasons of economy the Report has been reduced in size and the tables printed separately of comparative figures, between hospital and hospital, of expenditure under the chief headings are now condensed in each group into one table of analysis of cost of working. The total beds available in the 109 hospitals tabulated were 13,633, as against 12,463 in 1914, and the average occupancy 10,942 and 9,867 respectively. The reason for this condition may clearly be ascribed to the fact that in many institutions extra beds were put up in the wards and other parts of the hospital to meet the requirements of the wounded. The total new in-patients showed an increase of 8,406, and the out-patients fell off by nearly 100,000 as individual cases and 200,000 in attendances. Of the in-patients 28,645 were naval and military cases, and 128,865 civilians. The latter class were about 14,000 fewer than in 1914, and 15,000 fewer than in 1913. When one considers how many of the population who ordinarily would be hospital patients are absent from their homes, the above figures seem to demonstrate that the allocation of beds to naval and military patients does not, *prima facie*, appear to have caused great hardship to civilian users of hospitals; this, however, is not a question which can be answered by the quotation of these statistics alone, questions of length of stay, the standard of the severity of the case admitted, and other considerations would have to be carefully examined before a conclusion could be arrived at.

The total ordinary expenditure of the hospitals included in the statistics has risen from £1,142,938 in 1913 to £1,211,448 in 1914 and £1,342,127 in 1915, but during 1915 a much larger number of patients had to be maintained and, presumably, a larger staff of nurses and other workers. Mr. Maynard, in his summary of these figures, deducts from the total ordinary expenditure the payments made by authorities in aid of expenditure on naval and military patients, and expresses the result as "ordinary expenditure falling upon the normal sources of hospital income." This, we venture to think, is not an entirely fair way of presenting the figures: the calculation assumes that the hospitals would, if there had been no wounded, have filled the beds with free patients. It is probable that some of the beds used for our sailors and soldiers would have been occupied by contributing patients in ordinary times, and quite possible that, to meet extraordinary financial conditions, a system of patients paying towards the cost of maintenance according to their means would in many institutions have been started, a system which after the war is most likely to become fairly general.

